# An Automated Nanowell-Array Workflow for Quantitative Multiplexed Single-Cell Proteomics Sample Preparation at High Sensitivity

**DOI:** 10.1016/j.mcpro.2023.100665

**Published:** 2023-10-14

**Authors:** Claudia Ctortecka, David Hartlmayr, Anjali Seth, Sasha Mendjan, Guilhem Tourniaire, Namrata D. Udeshi, Steven A. Carr, Karl Mechtler

**Affiliations:** 1 Research Institute of Molecular Pathology (IMP), Vienna BioCenter (VBC), Vienna, Austria; 2 Broad Institute of MIT and Harvard, Cambridge, Massachusetts, USA; 3 Cellenion SASU, Lyon, France; 4 Institute of Molecular Biotechnology of the Austrian Academy of Sciences (IMBA), Vienna BioCenter (VBC), Vienna, Austria; 5 The Gregor Mendel Institute of Molecular Plant Biology of the Austrian Academy of Sciences (GMI), Vienna BioCenter (VBC), Vienna, Austria

**Keywords:** single-cell proteomics, proteoCHIP, automated sample preparation, isobaric peptide labeling, high field asymmetric waveform ion mobility spectrometry

## Abstract

Multiplexed and label-free mass spectrometry–based approaches with single-cell resolution have attributed surprising heterogeneity to presumed homogenous cell populations. Even though specialized experimental designs and instrumentation have demonstrated remarkable advances, the efficient sample preparation of single cells still lags. Here, we introduce the proteoCHIP, a universal option for single-cell proteomics sample preparation including multiplexed labeling up to 16-plex with high sensitivity and throughput. The automated processing using a commercial system combining single-cell isolation and picoliter dispensing, the cellenONE, reduces final sample volumes to low nanoliters submerged in a hexadecane layer simultaneously eliminating error-prone manual sample handling and overcoming evaporation. The specialized proteoCHIP design allows direct injection of single cells *via* a standard autosampler resulting in around 1500 protein groups per TMT10-plex with reduced or eliminated need for a carrier proteome. We evaluated the effect of wider precursor isolation windows at single-cell input levels and found that using 2 Da isolation windows increased overall sensitivity without significantly impacting interference. Using the dedicated mass spectrometry acquisition strategies detailed here, we identified on average close to 2000 proteins per TMT10-plex across 170 multiplexed single cells that readily distinguished human cell types. Overall, our workflow combines highly efficient sample preparation, chromatographic and ion mobility–based filtering, rapid wide-window data-dependent acquisition analysis, and intelligent data analysis for optimal multiplexed single-cell proteomics. This versatile and automated proteoCHIP-based sample preparation approach is sufficiently sensitive to drive biological applications of single-cell proteomics and can be readily adopted by proteomics laboratories.

## Highlights

• The proteoCHIP is designed for analyzing up to 576 single-cells per experiment.
• Multiplexed samples are pooled within the chip and inserted to any standard HPLC.
• Single-cell peptide input and ion mobility filtering reduce effects of co-isolation.
• Wide-window data-dependent acquisition identifies ∼2000 proteins per TMT10-plex.
• Unsupervised clustering separates cell-types across 170 multiplexed single-cells.

## Graphical Abstract

**Figure:**
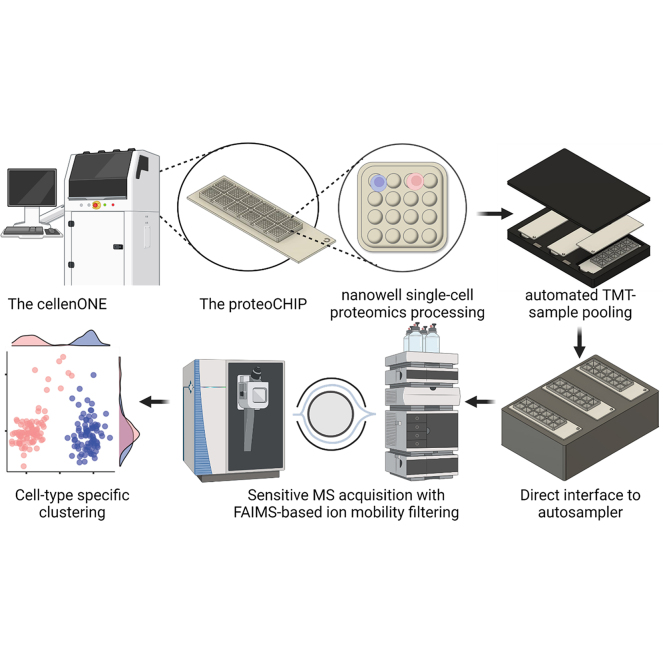

## In Brief

Multiplexed single-cell proteomics sample preparation using the proteoCHIP and the cellenONE minimizes adsorptive losses and variability, enabling quantification of 1500 protein groups per TMT10-plex without a carrier. Use of wider isolation windows (2 m/z) with FAIMS ion mobility filtering at single-cell input (∼150 pg) increases protein identifications with minimal negative effects. Our workflow identifies around 2000 proteins per TMT10-plex across 170 single cells, readily discriminating between two human cell types and begins to reveal potential differences in cell metabolism.

In recent years, single-cell analysis by transcriptional profiling has provided valuable insights into heterogeneous cell populations. However, proteins, and their posttranslational modifications, are the primary drivers of cellular identity and function. Therefore, complementing single-cell transcriptomics and genomics approaches with global protein analysis is essential. However, most protein profiling techniques with single-cell resolution still rely on the availability of antibodies. Continuous technological advances in sensitivity and accuracy of LC-MS/MS now enable single-cell proteomics (SCP) for hypothesis-free measurements.

However, analysis throughput, measurement variability, and, most importantly, sample preparation efficiency still lag behind comparable RNA-seq techniques. The combination of dedicated LC-MS/MS instrumentation with sensitive acquisition strategies for label-free single-cell analysis can be highly accurate but are limited in throughput and prone to peptide losses (1, 2). Throughput and sensitivity have been addressed through use of isobaric mass-tag labeling (*i.e.*, tandem mass tags [TMTs]), allowing peptides derived from individual cells to be uniquely barcoded and combined for simultaneous analysis and relative quantification by LC-MS/MS (3). TMT reagents are available in several multiplexing capacities enabling the analysis of up to 18 samples in one experiment (4, 5). This not only reduces the measurement time per single cell from about 25 min for label-free analysis to just 3.5 min but also increases the input material per sample. For multiplexed experiments, single cells are processed individually, TMT-labeled after tryptic digestion, and combined into one sample for mass spectrometry (MS) analysis. The nearly identical masses of the TMT labels allows for the simultaneous chromatographic elution of a peptide from all samples, therefore multiplying the precursor signal and contributing ions for peptide identification. Moreover, the differently equipped heavy isotopes of the TMT reagents enable relative quantification upon fragmentation in the MS. This approach was previously adopted to combine single cells with an abundant carrier spike for improved precursor selection, increased fragment ions for identification, and reduced peptide losses throughout the workflow (SCoPE-MS) (6). However, extreme carrier ratios (>20) were demonstrated to impair quantitative accuracy and possibly affect biological conclusions (7, 8).

Peptide losses during sample preparation were minimized with dedicated slide-based SCP sample preparation workflows, such as the nanodroplet processing in one-pot for trace samples (nanoPOTS) or nPOP (1, 9). These droplet-based sample preparations minimize sample volumes to less than 100 nl, which were automated *via* the cellenONE, a commercial liquid-dispensing instrument for cell isolation and single-cell preparation (9, 10, 11, 12). The latest generation nested nanoPOTS and nPOP smartly unify TMT channels with a microliter droplet on top of each sample array, greatly reducing manual sample handling. Nevertheless, to boost identifications, they combine the single cells with an abundant >10 ng bulk carrier and transfer the combined sample to a HPLC compatible vessel or rely on a custom-built nanoPOTS autosampler (12, 13).

To address these shortcomings, we have developed a versatile and automated workflow for preparation and analysis of multiplexed SCP samples within the cellenONE. Our optimized workflow combines highly efficient single-cell isolation, digestion, and TMT labeling using the proteoCHIP. Multiplexed samples are then analyzed by LC-MS/MS with ion mobility–based filtering and rapid, wider-isolation window data-dependent acquisition and intelligent data analysis (*i.e.*, CHIMERYS). We present cross-contamination–free processing of up to 592 single cells per experiment without any manual sample handling and a direct interface with standard autosamplers for accurate protein identification and quantification. The commercial availability of all components makes the entire approach readily adoptable and can be implemented by most proteomics laboratories.

## Experimental Procedures

### Mammalian Cell Culture

HeLa, S2, and HEK-293T cells were cultured at 37 °C and 5% CO_2_ in Dulbecco's modified Eagle's medium supplemented with 10% fetal bovine serum and 1× penicillin-streptomycin (P0781-100ML, Sigma-Aldrich) and L-Glut (25030-024, Thermo Fisher Scientific). After trypsinization (0.05% Trypsin-EDTA 1×, 25300-054, Sigma-Aldrich), cells were pelleted, washed 3× with PBS, and directly used for single-cell experiments.

### Bulk Sample Preparation

Cell pellets were lysed using a methanol/chloroform/water solution (4:1:3), briefly sonicated, and dried in a vacuum concentrator. The pellets were then resuspended in 8 M urea in 10 mM HCl, adjusted to 200 mM Tris/HCl pH 8.0, reduced using DTT (50 mM, 37 °C, 30 min), and alkylated with iodoacetamide (40 mM, 30 min at room temperature (RT)). The samples were diluted to a final concentration of 4 M urea in 100 mM Tris/HCl pH 8.0 and digested with LysC (Wako, enzyme/protein 1:100) for 3 h at 37 °C. Samples were adjusted to pH 2 with 10% trifluoroacetic acid (TFA), desalted using C18 solid-phase extraction cartridges (SPE, C18 Sep-pak, 200 mg Waters), and eluted with 40% acetonitrile (ACN) in 0.1% TFA. The solid-phase extraction cartridge eluate was dried to completeness in a vacuum centrifuge and TMT-labeled according to the manufacturer’s instructions. Briefly, samples were labeled in 100 mM triethylamine bicarbonate (TEAB) and 10% ACN for 1 h at RT. The unreacted TMT reagent was quenched with 5% hydroxylamine/HCl for 20 min at RT and subsequently mixed at equimolar levels corresponding to each sample pool (two-proteome mixture). To exclude any label-specific effects, two mixes were compiled of HeLa cell lysate channels: 126, 127C, 128C, 129C, 130C; S2 cell lysate channels: 127N, 128N, 129N, 130N, 131 or S2 cell lysate channels: 126, 127C, 128C, 129C, 130C; and HeLa cell lysate channels: 127N, 128N, 129N, 130N, 131.

### Single-Cell Sample Preparation With the cellenONE

For single-cell sample preparation, 40 nl lysis buffer (0.2% N-dodecyl-β-D-maltoside (D4641-500MG, Sigma-Aldrich), 100 mM TEAB (17902-500ML, Fluka Analytical), 10 ng/μl trypsin (Promega Gold, V5280, Promega)) was dispensed into each well of the proteoCHIP using the cellenONE (Cellenion) at elevated humidity (80%). After single-cell deposition at 75% humidity (gated for cell diameter min. 22 μm and max. 33 μm, circularity 1.1, and elongation 1.84), cells within the mastermix were submerged with a layer of Hexadecane (H6703-100ML, Sigma-Aldrich). The chip was then incubated at 50 °C for 2 h, directly on the heating deck inside the cellenONE at 80% humidity for lysis and digestion. For TMT labeling, 60 nl of 100 mM TEAB and 100 nl of either 10 mM TMT10 or TMTpro in anhydrous ACN was added to the respective wells and incubated for 1 h at RT and 80% humidity. To eliminate batch effects of TMT label assignments, all multicell type experiments were performed with a label switch and label randomization (*i.e.*, opposing label assignment or scrambled). TMT was subsequently quenched with 50 nl 0.5% hydroxylamine (90115, Thermo Fisher Scientific) and 3% hydrochloric acid.

TMT-labeled single-cell samples were subsequently pooled *via* centrifugation at 1500 rpm for 2 min into the funnel part of the proteoCHIP enabling direct injection to the LC-MS/MS system. Alternatively, labeled cells may be transferred to 0.2 ml PCR-tubes coated with 0.0003% polyethylene glycol (PEG) (95172-250G-F, Sigma-Aldrich) and 0.1% TFA with an approximate final volume of 3.5 μl. Samples presented within this manuscript have been transferred and injected from 0.2 ml PCR tubes. The addition of 0.0003% PEG to the autosampler vials improves peptide recovery over elongated storage times, alternatively the samples can be supplemented with 5% dimethyl sulfoxide, as previously described (14, 15, 16). The final sample volume was increased to approximately 3.5 μl as described above to ensure accurate injection with the below described Ultimate 3000 RSLC-nano ultra-high-performance liquid chromatography (UHPLC). Dedicated autosamplers or more recent generations of HPLCs now are capable of accurately and reproducibly injecting such nanoliter samples (17, 18). In the autosampler, both the funnel part of the proteoCHIP and PCR tubes are sealed with aluminum foil or a silicone matte in the respective holders to minimize sample evaporation for 12 h at 4 °C. Prolonged storage (*i.e.*, >24 h) in the autosampler reduces sample volumes by 40 to 50%, which needs to be considered for accurate injection. All carrier channels were sorted within the cellenONE and prepared identical to the single-cell samples.

### LC-MS/MS Analysis

Samples were measured on an Orbitrap Exploris 480 Mass Spectrometer (Thermo Fisher Scientific) with a reversed phase Thermo Fisher Scientific Ultimate 3000 RLSC-nano UHPLC system coupled *via* a Nanospray Flex ion source equipped with field-asymmetric ion mobility spectrometry (FAIMS) (operated at −50 compensation voltage [CV]). Labeled peptides were first trapped on an Acclaim PepMap 100 C18 precolumn (5 μM, 0.3 mm × 5 mm, Thermo Fisher Scientific) and eluted to the analytical column nanoEase M/Z Peptide BEH C18 column (130 Å, 1.7 μm, 75 μm × 150 mm, Waters) developing a two-step solvent gradient ranging from 2 to 20% over 45 min and 20 to 32% ACN in 0.08% formic acid within 15 min, at a flow rate of 250 nl/min.

For isolation window width evaluations (supplemental Fig. S4), we utilized a Thermo Fisher Scientific Vanquish Neo UHPLC system with direct injection setup and a capillary column (PicoFrit with 10 μm tip opening/75 μm diameter, New Objective, PF360-75-10-N-5) packed in-house with 25 cm C18 silica material (1.9 μm ReproSil-Pur C18-AQ medium, Dr Maisch GmbH, r119.aq). The capillary column was heated to 50 °C to reduce backpressure. Samples were loaded using pressure control fast loading up to 600 bar, separated with a two-step solvent gradient ranging from 2 to 20% solvent B (0.1% formic acid in ACN) over 45 min and 20 to 45% solvent B within 20 min at a flow rate of 200 nl/min.

Full MS data were acquired in a range of 375 to 1200 m/z with a maximum automatic gain control (AGC) target of 3e6 and automatic inject time at 120,000 resolution. Top ten multiply charged precursors (2–5) over a minimum intensity of 5e3 were isolated using a 2 m/z isolation window. Tandem mass spectrometry (MS/MS) scans were acquired at a resolution of 60,000 at a fixed first mass of 110 m/z with a maximum AGC target of 1e5 or injection time (IT) of 118 ms. For method evaluation, a range of isolation windows were tested including 0.4, 0.7, 1, 1.5, 2, and 3 m/z, as indicated. Previously isolated precursors were subsequently excluded from fragmentation with a dynamic exclusion of 120 s. TMT10 precursors were fragmented at a normalized collision energy of 34 and TMTpro at a normalized collision energy of 32.

### Data Analysis

Peptide identification was performed using CHIMERYS within the Proteome Discoverer 3.0 environment against the human reference proteome sequence database (UniProt; version: 2021-06-30). Searches were performed for specific tryptic peptides in the range of 7 to 30 amino acids with maximum two missed cleavages and maximum three modifications per peptide. Fragment mass tolerance was set to 20 ppm with maximum 15 candidates per MS/MS spectrum. Oxidation at methionine was set as variable modifications and the respective TMT reagents were selected as fixed modification. Peptide spectrum match (PSM), peptide, and protein groups were filtered with a false discovery rate of 1% using percolator. Reported PSMs or peptide identifications (IDs) are required to present at least one reporter ion across all single-cell channels per analytical run. Signal to noise (S/N) and intensity of reporter ions were extracted using the Proteome Discoverer node Hyperplex, developed in the Mechtler lab (freely available: pd-nodes.org) at 10 ppm, and assigned to the top hit per MS/MS spectrum for quantification. Postprocessing was performed in the R environment if not indicated otherwise. For quantification, PSMs were summed to peptides and protein groups. Single-cell reporter ion intensities are normalized to their sample loading within each analytical run. For HeLa/HEK, the raw reporter ion intensities were log_2_ transformed; protein groups with less than 70% missing data across the entire dataset were imputed with random values from a normal distribution shifted into the noise. All protein groups with more than 70% missing data across the dataset were removed from subsequent analysis. The reporter ion intensities were then quantile normalized, batch corrected using ComBat for the analytical run, and the TMT channel. For volcano plots, peptide quantities were summed to proteins and plotted using the Perseus interface (19, 20, 21). Venn diagrams are based on distinct peptide sequences and are calculated using BioVenn (20). Grand average of hydropathy scores were calculated for every distinct peptide sequence identified from the respective condition, based on the amino acid hydropathy scores (22). “Outlier” cells (supplemental Fig. S4) are defined as samples beyond 25th or 75th percentile ± 1.5∗interquartile range of principal component (PC)2. Pathway analysis of proteins with highest fold change between “non-outlier” and “outlier” samples supplemental Fig. S4 was performed within String-db v12 (https://string-db.org/) using the KEGG database.

### Experimental Design and Statistical Rationale

Comparison of TMT10 to TMTpro (16-plex) with no-carrier and 20× carrier were comprised of one sorted single-HeLa cell per channel except for “20× carrier samples” using one channel (126) for 20 sorted HeLa cells, leaving the adjacent 127C TMT-channel empty to minimize isobaric interference. We acquired LC-MS/MS data for 25 TMT10 no-carrier (250 cells); seven TMT10 with 20× carrier (56 cells); five TMTpro no-carrier (80 cells) and 14 TMTpro with 20× carrier (196 cells). For co-isolation evaluation, we combined bulk digested and TMT10-labeled HeLa and *Drosophila melanogaster* S2 samples. For this, we used five TMT channels per species and mixed them at a 1:1 ratio at a final on column peptide amount of 100, 10, and 1 ng per analytical run. These TMT mixes were prepared on three separate days and injected in duplicates. Similarly, for the HeLa HEK comparison without a carrier, we used five TMT channels per cell line, labeled with all available TMT10 tags and acquired 17 analytical runs (170 cells). These 17 analytical runs entail three proteoCHIP batches, comprised of three biological replicates (*i.e.*, cell passages), proteoCHIP workflows, and acquisition dates (supplemental Fig. S4). We prepared five TMT10-multiplexed samples for batch 1 and six TMT10-multiplexed samples for batches 2 and 3. For the six HeLa HEK with 20× carrier TMT10 samples, we included a carrier labeled with the 131 TMT channel, leaving the 130N label empty to minimize isobaric interference and eight single cells (*i.e.*, four HeLa and four HEK-293 cells). The six TMT10 HeLa HEK with 20× carrier samples were prepared along with batch 1 (supplemental Fig. S4) and the carrier was comprised of ten HeLa and ten HEK-293 cells to include sample-specific peptide species and equally enhance precursor signal (total of 48 cells). Combining no-carrier and 20× carrier samples, we acquired 218 single HeLa and HEK-293 cells in three biological replicates.

## Results

### Multiplexed SCP Sample Preparation With the proteoCHIP

**Fig. 1:**
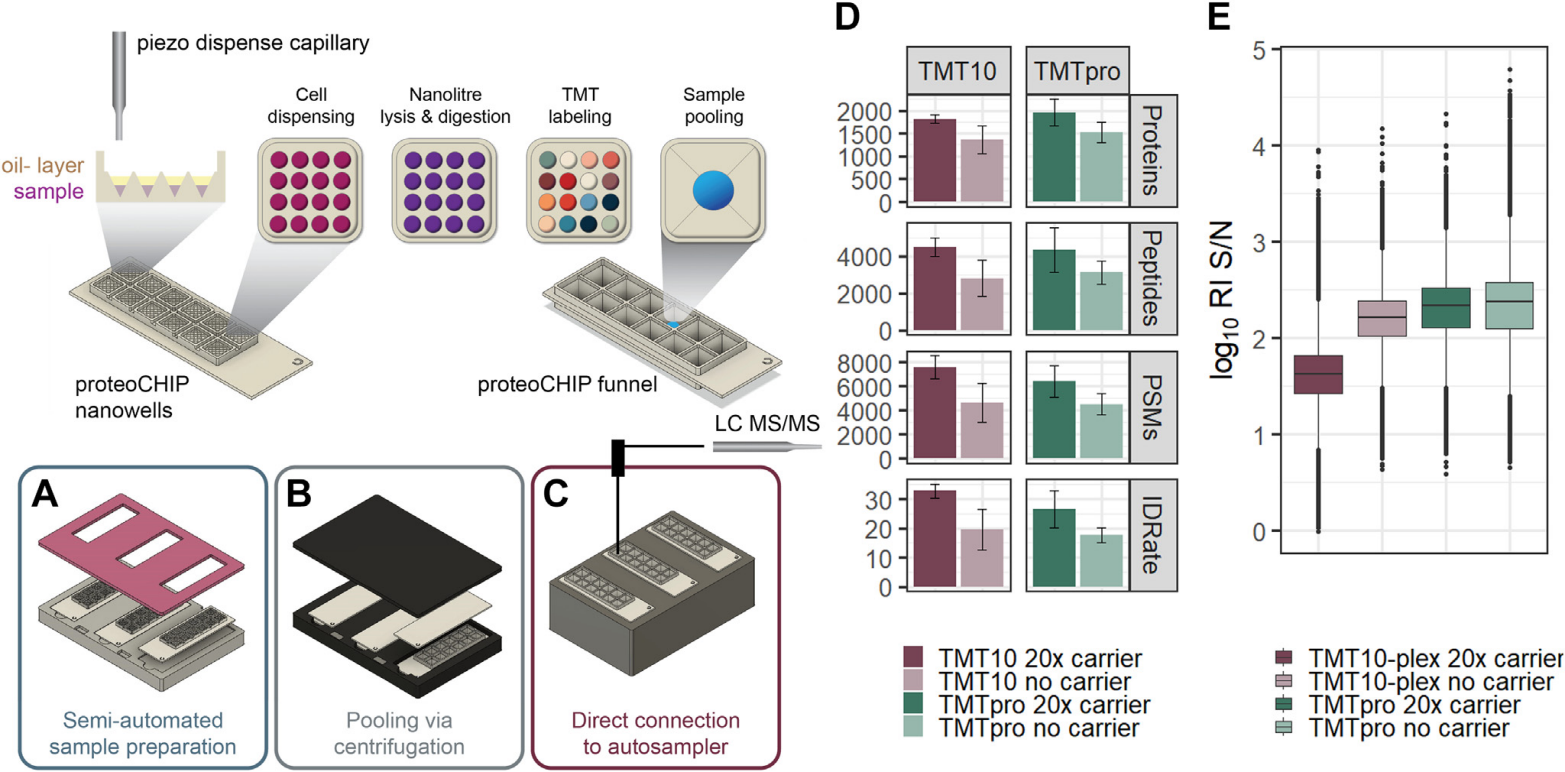
**The proteoCHIP multiplexing workflow for TMT10 and TMTpro.***A*, up to 16 nanowells/single cells per TMT set are prepared inside the cellenONE, (*B*) automatically combined *via* benchtop centrifugation, and (*C*) directly interfaced with a standard autosampler. *D*, identified proteins, peptides, PSMs, and ID-rate and (*E*) log_10_ reporter ion S/N of single cells labeled with TMT10 (*red*) or TMTpro (*green*) at 20× or no-carrier. A total of 250 single cells were processed for TMT10 no-carrier, while 56 were included for TMT10 20× carrier. For TMTpro with no-carrier, 80 single cells were processed, while for TMTpro 20× carrier, 196 single cells were used. PSM, peptide spectral match; RI, reporter ion; S/N, signal to noise; TMT, tandem mass tag.

Here, we introduce the proteoCHIP as a viable option for automated SCP sample preparation within the cellenONE, a robotic platform combining single-cell isolation and picoliter dispensing. The nanowell part of the proteoCHIP is designed to process 192 single-cells simultaneously (12 multiplexed sets of 16 samples) and up to 576 single-cells per experiment (*i.e.*, three proteoCHIPs within the holder; Fig. 1, *A* and *B*). The proteoCHIP is the size of a standard microscopy slide. It is composed of polytetrafluoroethylene which overcomes peptide losses to plastic or glassware with similar grand average of hydropathy indices compared to dedicated low-binding and PEG-coated autosampler vials (supplemental Fig. S1) (16, 23). The entire proteoCHIP sample preparation workflow, including lysis, digestion, and TMT-labeling, is performed within the cellenONE and has been automated by Cellenion (see Single-cell sample preparation within the cellenONE in the Experimental Procedures for details). Throughout the initial setup, the user is prompted with tasks to insert the chip, add the mastermix and the cells at indicated positions/wells. When these steps are completed, the cells are incubated for a predefined time, which is followed by the addition of TMT in ACN to the cellenONE. After digestion and labeling with TMT reagents for quantification, the labeled peptides in each plex are pooled automatically *via* a benchtop centrifuge into the funnel of the proteoCHIP that is directly interfaced with a standard HPLC autosampler (Fig. 1*C*). To address the critical issue of evaporation of low nanoliter sample volumes, we employ an oil layer to maintain constant enzyme and chemical concentration. Hexadecane was chosen as it solidifies at autosampler temperatures (approx. 4–10 °C) and is therefore not injected along with the single-cell sample to prevent interference with either HPLC injection or chromatography. The oil layer slows down evaporation preventing the nanoliter sample from drying completely during incubation. After pooling samples in the proteoCHIP funnel, the sample volume exceeds oil coverage; hence for longer storage in the autosampler, the proteoCHIP funnel is covered with aluminum foil or silicone mats to avoid evaporation up to 12 h at 4 °C. While our aim is to minimize reaction volumes to approximately 20 to 150 nl for single-cell workflows, the proteoCHIP nanowells can hold up to 600 nl without cross-contamination of the samples if higher reaction volumes are necessary for the workflow (data not shown).

We reasoned that the integrative proteoCHIP design could reduce the need for a carrier channel to generate protein IDs comparable to other techniques (12, 24). We and others have evaluated the use of a maximum of 20 carrier cells to boost sensitivity, reduce missing data, and limit the impact of carrier-induced reporter ion signal suppression (7, 8, 11, 12, 25, 26, 27). To test the applicability of the proteoCHIP sample preparation with all TMT reagents available at that time, we processed multiple SCP batches with both TMT10 and TMTpro (16-plex), with and without a 20× carrier to compare results obtained using the two distinct TMT reagent sets at optimized conditions. Using TMT10, 250 single HeLa cells without a carrier were analyzed in 25 injections and 56 single HeLa cells with a 20× carrier were acquired in seven analytical runs. A median of 1947 protein groups based on 4492 peptides were identified per analytical run using a 20× carrier, while the samples without a carrier yielded 1489 median protein groups based on 2807 peptides (Fig. 1*D*). The union of all 306 single cells analyzed by TMT10 with and without a carrier yielded 2913 protein groups based on 11,791 peptides. For TMTpro, 80 single cells were analyzed without a carrier in five analytical runs, while 196 single cells were acquired with a 20× carrier in 14 injections. A median of 1957 protein groups from 4354 peptides per analytical run were observed with the 20× carrier, while a median of 1519 protein groups based on 3123 peptides were obtained without a carrier. Across all 19 TMTpro-labeled single-cell injections, combining carrier and no-carrier samples, we identified over 3674 protein groups from 14,830 peptides.

Interestingly, the number of proteins identified using TMTpro *versus* TMT10, with and without use of a carrier channel, were comparable but the median reporter ion S/N was higher in the TMTpro-labeled samples (Fig. 1*E*) (7). This could be due to the different fragmentation behavior of the longer TMTpro tag or the numerical increase of single-cell channels per TMT-plex (4, 5). Importantly, single-cell reporter ion S/N is reduced by 52% for TMT10 and 25% for TMTpro in the 20× carrier *versus* no-carrier samples (Fig. 1*E*). Despite the low ratio between the carrier to single-cells (*i.e.*, 20×), we speculate that for TMT10 experiments, the larger proportion of ions sampled from the carrier relative to the single-cells compresses the single-cell reporter ion signals to a greater extent than TMTpro experiments (Fig. 1*E*). As expected, peptides that are uniquely identified in the carrier samples are generally lower in abundance than peptides that are uniquely identified in no-carrier samples for both TMT10 and TMTpro experiments (supplemental Fig. S2, *A* and *B*). This indicates that the carrier boosts low abundant peptides, but reporter ion ratio compression generally results in lower S/N than the no-carrier samples (Fig. 1*E* and supplemental Fig. S2, *A* and *B*). Again, high reporter ion signal has been shown to parallel quantitative accuracy in MS2-based TMT quantification (7, 8). Therefore, although the carrier undoubtedly boosts identifications in SCP, we suggest keeping the carrier ratio to a minimum due to the reporter ion S/N compression (Fig. 1, *D* and *E*) (7, 8, 11). Additionally, TMTpro demonstrates reduced reporter ion S/N compression at comparable peptide and protein identifications over TMT10 in multiplexed SCP experiments. This suggests that a larger relative proportion of TMT channels containing single-cell relative to the single-carrier channel is beneficial for quantitative accuracy.

**Fig. 2:**
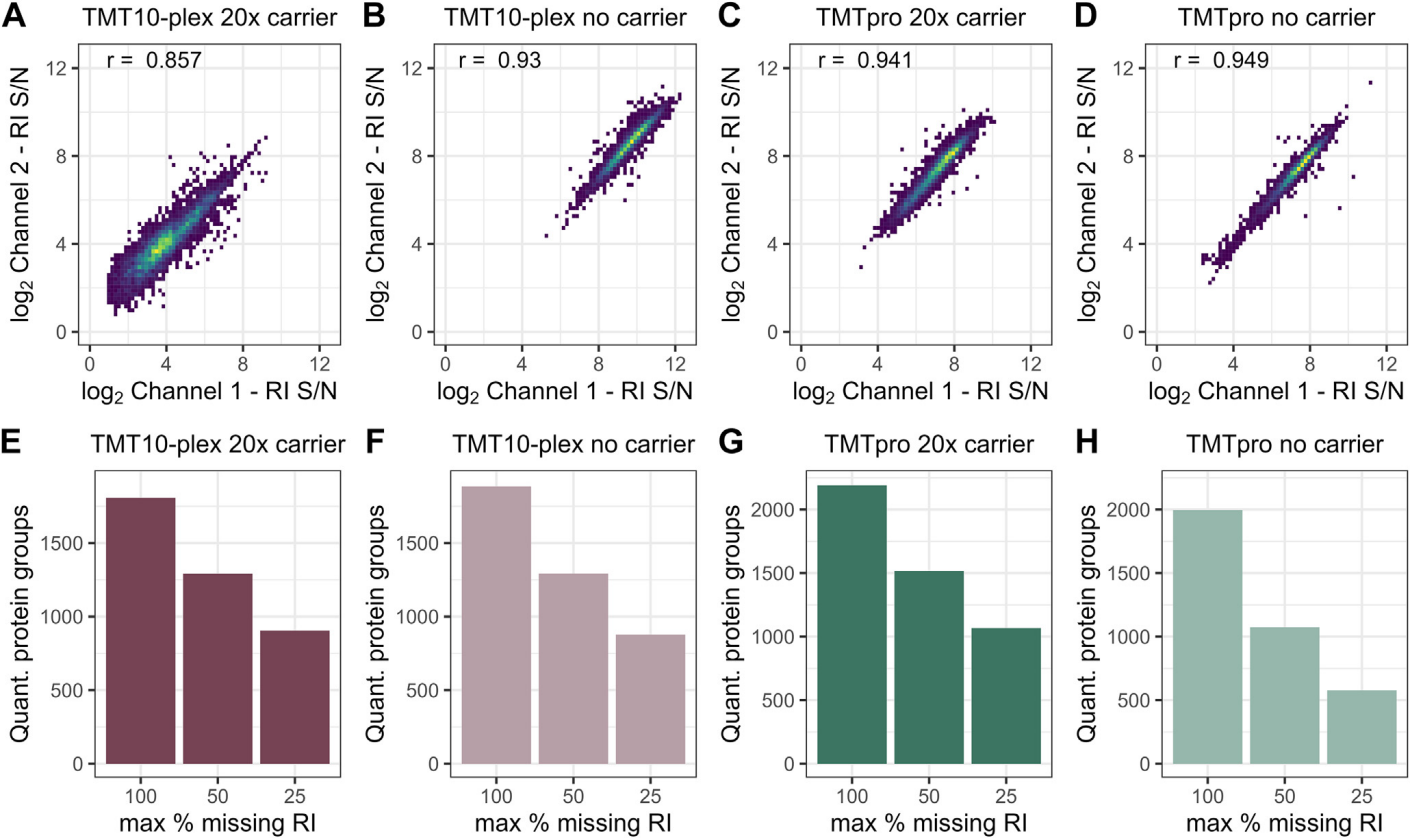
**Data completeness and reproducibility of multiplexed single-cell proteomes with TMT10 and TMTpro reagents at different sorted carrier ratios.** Log_2_ reporter ion S/N correlation of two single-cell samples within one TMTplex for (*A*) TMT10 with a 20× carrier channel or (*B*) no-carrier, (*C*) TMTpro with a 20× carrier channel, or (*D*) no-carrier; r = Pearson correlation estimate. Cumulative missing reporter ions per quantified protein across three analytical runs for (*E*) TMT10 with a 20× carrier channel or (*F*) no-carrier, (*G*) TMTpro with a 20× carrier channel, or (*H*) no-carrier samples across three injections per condition. RI, reporter ion; S/N, signal to noise; TMT, tandem mass tag.

To evaluate quantitative reproducibility and data quality, we analyzed missing reporter ion signal per PSM and cumulative missing data across multiple analytical runs. For both TMT labeling reagents, with or without a carrier, the reporter ion S/N of any two individual sample channels correlated well (r > 0.88), with over 90% of all PSMs having a maximum of one quantitative channel missing across all experimental setups (Fig. 2, *A*–*D*). While this level of quantitative comprehensiveness in single-cell analyses is better than previous reports of multiplexed SCP (12, 24), precursor stochasticity accumulated distinct sets of identified precursors across analytical runs, thus decreasing the sample overlap across three analytical runs (Fig. 2, *E*–*H*). We speculate that the TMT labels and other reagents contribute to background signal and chemical noise, which interferes with precursor selection and MS/MS identification. Data aggregation of three analytical runs for both reagents (*i.e.*, 30–48 single cells) reduces common protein identifications by 50% without matching between runs (Fig. 2, *E*–*H*, third bar to right in each plot), as previously reported by others (12, 24). Despite excellent data quality, standard data-dependent acquisition (DDA) accumulates missing data in large sample cohorts reducing replicate overlap and requiring data imputation, which has to be considered prior to any SCP experiment (28, 29).

### The Impact of Isolation Width on Isobaric Label Quantification Accuracy Is Input-Dependent

High-input TMT studies (*e.g.*, microgram peptide input per channel) generally employ precursor isolation widths of <1 m/z. However, prior ultra-low input and single-cell reports using either Orbitrap or trapped ion mobility spectrometry time-of-flight instruments described accurate TMT-based quantification in DDA or data independent acquisition utilizing >1 m/z isolation windows to increase sensitivity (8, 29, 30, 31). In the analyses we describe above, we employed an isolation window of 2 m/z, a maximum IT of 118 ms and an AGC target of 1e5 to increase the number of isolated prospective peptide precursor ions without impeding cycle time. Before employing this approach for biological studies, we sought to understand the effect that widening the isolation window has on the quantitative accuracy in low input or single-cell analyses. We hypothesized that coisolation of precursors using a wider isolation window would be minimized given our use of FAIMS-based ion mobility filtering, together with a 60-min effective liquid chromatography gradient to analyze samples having less than a nanogram of peptide per analytical run. Other multiplexed single-cell studies have utilized >200 ms IT to increase precursor ion sampling, but as our aim was to simultaneously reduce precursor stochasticity across large cohorts, we instead used a shorter IT (118 ms = transient time) in combination with a wider isolation window (32, 33).

To evaluate the impact of coisolation on the identification and quantification of peptides at both low- and high-input levels, we generated a two-proteome mix of bulk-digested HeLa (human) and S2, *D. melanogaster* (dros.) cell lines. We isobarically labeled aliquots of both digests with all available TMT10 tags, diluted them to 100 pg (*i.e.*, approximate peptide yield of a single mammalian cell), 1 or 10 ng peptide per channel, and combined them to TMT10-plexes (34). We then injected a total 1 ng (100 pg per channel), 10 ng (1 ng per channel), and 100 ng (10 ng per channel) per analytical run. Each sample was analyzed with varying isolation windows of 0.4, 0.7, 1, 1.5, 2, and 3 m/z on the Orbitrap Exploris 480 using FAIMS at a CV of −50. We and others utilize a single FAIMS CV when measuring limited sample amounts in contrast to most higher-input FAIMS studies combining multiple CVs to reduce sample complexity (15, 35, 36, 37). The use of only one CV (*i.e.*, CV −50) reduces singly charged background noise while increasing the signal detected for multiply charged precursors without detrimental effects on cycle times (29, 35). Data were analyzed using CHIMERYS and the Proteome Discoverer node, Hyperplex, developed in the Mechtler lab for evaluation of reporter ion intensity and S/N (38, 39).

**Fig. 3:**
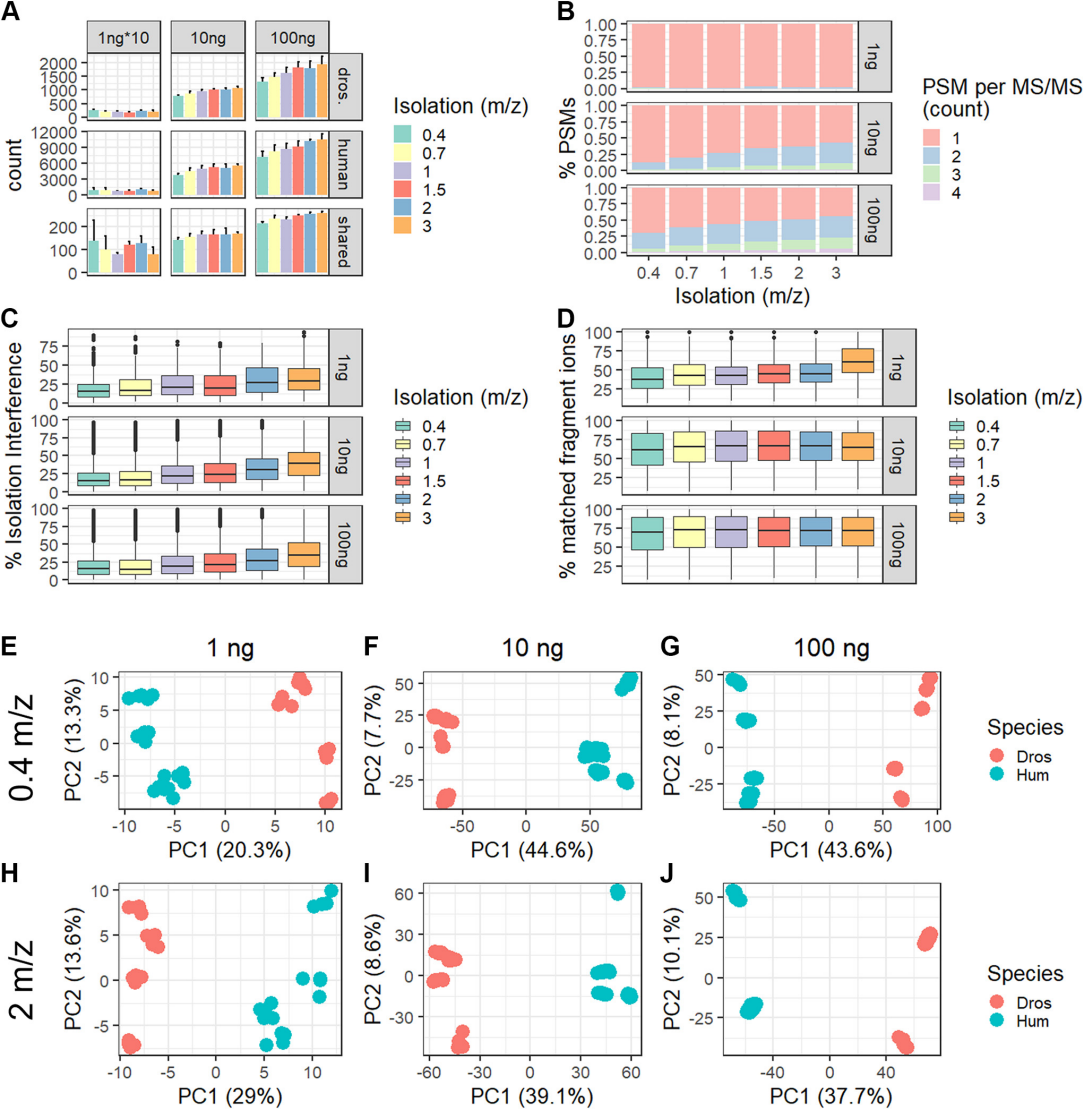
**Large isolation windows do not result in frequent chimeric spectra at single-cell peptide input with FAIMS-based ion mobility filtering.***A*, protein identifications assigned per species and isolation windows (0.4, 0.7, 1, 1.5, 2, or 3 m/z) across 1∗10, 10, or 100 ng total sample input. *B*, % PSMs identified from chimeric MS/MS scans (>2 peptides identified per MS/MS scan) per isolation window (0.4, 0.7, 1, 1.5, 2, or 3 m/z) across 1, 10, or 100 ng total sample input. *C*, % isolation interference and (*D*) % matched fragment ions per PSM. Colors indicate isolation window (0.4, 0.7, 1, 1.5, 2, or 3 m/z) across 1, 10, or 100 ng total sample input. Cell type–specific separation by PCA on the peptide level using a 0.4 m/z isolation window with (*E*) 1, (*F*) 10, or (*G*) 100 ng total sample input. PCA of two-proteome mix isolated with 2 m/z at (*H*) 1, (*I*) 10, or (*J*) 100 ng peptide input. Colors indicate different species; each data point represents one sample. FAIMS, field-asymmetric ion mobility spectrometry; MS/MS, tandem mass spectrometry; PCA, principal component analysis; PSM, peptide spectral match.

The evaluation of coisolation on quantification is focused on species-specific and shared peptide sequences. While peptide and protein identifications across species remained relatively constant with increasing isolation window size at 1 ng loading (*i.e.*, 100 pg per channel), we observed ∼25% higher peptide and protein identifications at 3 m/z isolation window than 0.4 m/z at 10 and 100 ng total peptide input per TMT-plex (Fig. 3*A*). At the 1 ng total sample input, we mostly identified one peptide per MS/MS scan across all isolation widths (Fig. 3*B* and supplemental Fig. S3*A*). However, with 10-fold increased input, more than 40% of all MS/MS scans are chimeric at 3 m/z isolation width. Similarly, at 100 ng total peptide input, more than 60% of all acquired MS/MS scans give rise to two or more peptide identifications (Fig. 3*B* and supplemental Fig. S3*A*). Moreover, the % isolation interference per scan, which we defined by the presence of other peaks in the isolation window other than the triggered peptide precursor, reflects a similar trend (Fig. 3*C*). Even though we observe a small and gradual increase of interference using wider isolation windows starting at 1 ng peptide input, this rarely resulted in the identification of two peptides per MS/MS scan (Fig. 3*B* and supplemental Fig. S3*A*). In this study, wider isolation windows resulted in higher % matched fragment ions per peptide, thereby increasing the confidence of any identification. This increase in matched fragment ions with wider isolation window widths was only observed at 1 ng peptide input, while 10 and 100 ng consistently show around 65% or 71% of matched fragment ions, respectively (Fig. 3*D*). In detail, at 1 ng peptide level input with 0.4 m/z isolation window, 38% of fragment ions were matched, which increased to 46% at 1.5 m/z and up to 61% at 3 m/z (Fig. 3*D*). Moreover, the average reporter ion S/N per PSM increased by >50% in S/N going from 0.4 to 3 m/z for all input levels across all MS/MS spectra (supplemental Fig. S3*B*). Notably, chimeric spectra (≥2 peptides per MS/MS scan) only showed 30% increased reporter ion S/N relative to nonchimeric spectra independent of the isolation window width (supplemental Fig. S3*B*). As expected, wider isolation windows result in more coisolation and therefore more isolation interference and chimeric spectra at >10 ng peptide input. We suspect that the combination of relatively long chromatographic gradients and FAIMS filtering resulted in the gradual decrease of chimeric spectra with input amount, ultimately yielding higher sensitivity with close to no chimeric spectra at single-cell equivalents of 100 pg peptide per channel.

Based on these observations, we evaluated quantification accuracy *via* the ability to differentiate two proteomes across all isolation widths and input levels. Due to both the continuous increase in identification but also isolation interference with wider isolation windows, we decided to continue with a maximum of 2 m/z width (Fig. 3, *A*–*D*). For quantitative evaluation, we combined three batches (*i.e.*, bulk dilutions) of our two-proteome mix at a total peptide input of 1, 10, or 100 ng for 0.4 and 2.0 m/z isolation windows and subjected them to principal component analysis (Fig. 3, *E*–*J*). For this, we merge peptide identifications, filter for >70% data completeness across all analytical runs, and display each channel as one sample across PC 1 and 2 (Fig. 3*J*). At all input levels, samples cluster mostly by species (PC1) rather than batches (PC2). Even though the PC1-explained variance separating the two species increases 2-fold from 1 ng to 100 ng, the driving difference for all conditions is species-specific. Interestingly, at 10 ng and 100 ng total peptide input, the explained variance of PC1 decreases with increasing isolation window, while the opposite trend is observed for 1 ng. This indicates that at higher peptide input, the co-isolation negatively affects the species-specific separation, which is not the case at 1 ng (Fig. 3, *E*–*J*). Of note, batch-specific separation is most apparent at 100 ng peptide input with a 2 m/z isolation window, which we speculate is due to increased precursor co-isolation (Fig. 3*J*). Aiming to artificially increase batch variability, we acquired the third batch of our two-proteome mix on a different Exploris 480 and liquid chromatography instrument including another column. Even with increased batch variability, sample identity remains the largest proportion of variance across all input levels and isolation windows, included in this study (Fig. 3, *E*–*J*). Based on this, we are confident that acquisition of TMT-labeled single-cell data using an isolation width of 2 m/z in combination with FAIMS allows for profiling at single-cell peptide level inputs with limited precursor stochasticity and minimal effect on quantitative accuracy.

### Cell Type–Specific Clustering of Two Human Cell Lines Based on Their Single-Cell Proteome

**Fig. 4:**
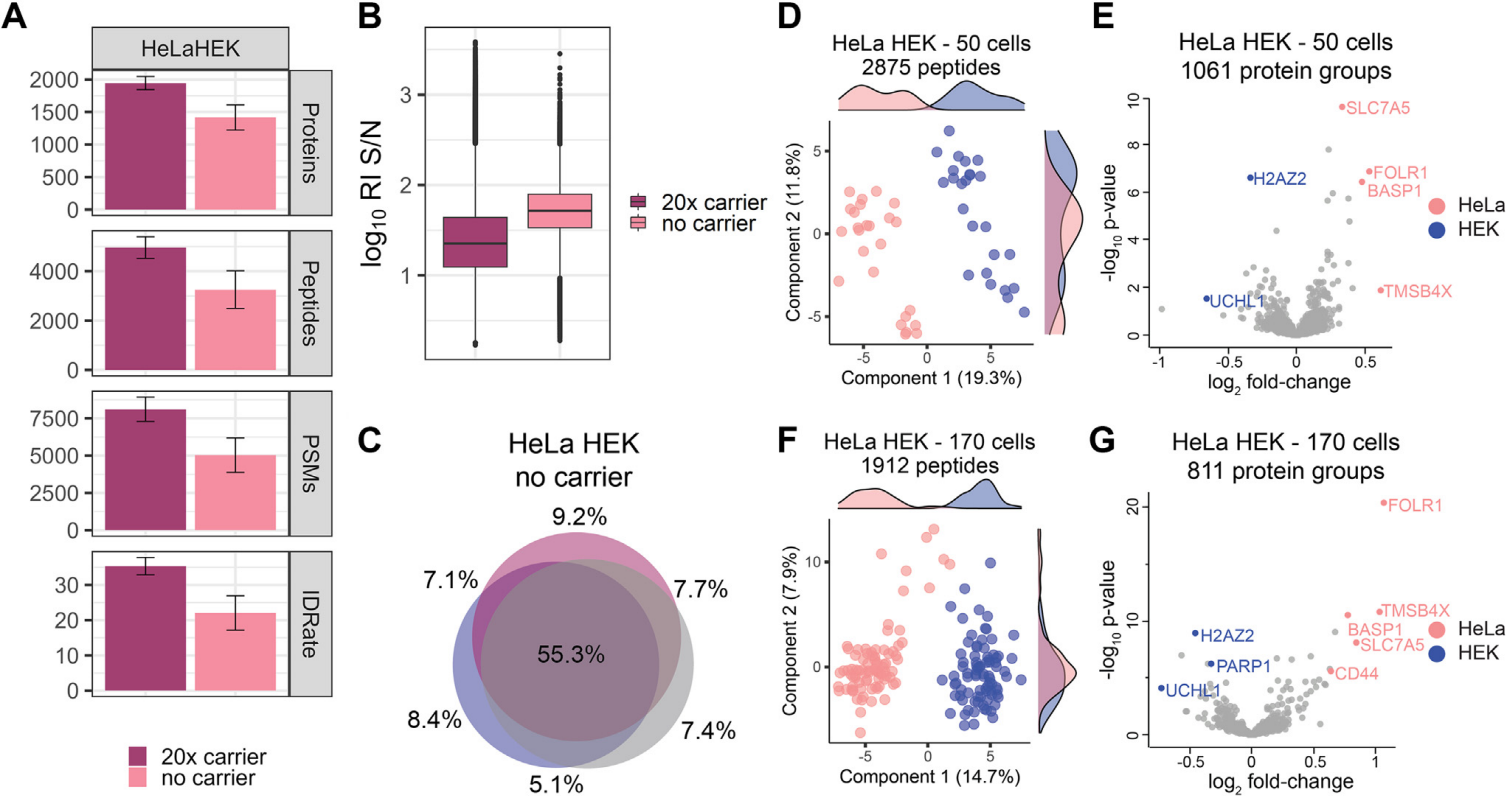
**Comparison of HeLa and HEK-293 single-cell proteomes.***A*, protein groups, peptides, PSMs, MS/MS scans, ID-rate and (*B*) reporter ion S/N of HeLa/HEK-293 samples with 20× sorted and no-carrier. *C*, distinct peptide sequence overlap across three analytical runs of HeLa/HEK-293 no-carrier samples. Fifty single-cell (*D*) PCA with Kernel density estimates and (*E*) differential protein abundance or 170 single-cell (*F*) PCA with Kernel density estimates and (*G*) distinct proteins of HeLa (*pink*) and HEK-293 (*blue*). For volcano plots, log_2_ fold change and −log_10_*p*-value is shown. MS/MS, tandem mass spectrometry; PCA, principal component analysis; PSM, peptide spectral match; RI, reporter ion; S/N, signal to noise.

To benchmark our proteoCHIP-based sample preparation and data acquisition approach, we aimed at differentiating two similarly sized human cell types, HeLa and HEK-293, based on their proteome (supplemental Figs. S4 and S5*A*). HeLa and HEK-293 cells were sorted, lysed, digested, labeled with TMT10 reagents, and combined with or without a 20× mixed carrier channel comprised of 50% HeLa and 50% HEK-293 sorted cells (supplemental Fig. S4). In detail, for “no-carrier” samples, we combined five HeLa and five HEK-293 cells per analytical run, while for the 20× mixed carrier samples, one channel was used for the carrier (126), leaving the adjacent 127C empty to reduce isobaric interference. A total of 218 TMT10-labeled single cells were analyzed across 23 analytical runs and we identified cumulatively 2598 proteins and 9375 peptides across all single cells (Fig. 4*A*). Reporter ion intensities were highly similar for both cell types, lending confidence that quantitative differences observed in the proteomes of the two cell lines would reflect distinction in their constitution rather than differences in sample input (Fig. 4*B*). In detail, the median yield obtained from six HeLa/HEK-293 TMT10 runs using a 20× carrier channel was 1944 proteins from 4960 peptides, while the 17 no-carrier samples yielded 1416 proteins from 3256 peptides per analytical run (Fig. 4*A*). Similar to the HeLa-only samples, a 20× sorted carrier compressed the reporter ion S/N by 57% while protein IDs increased by 19% (Figs. 1*E* and 4, *A* and *B*). Moreover, both the 20× sorted and no-carrier samples demonstrated comparable replicate overlap; therefore, we proceeded without the use of a carrier in subsequent studies below for enhanced quantitative data quality (Fig. 4*C* and supplemental Fig. S5*C*).

To minimize technical variability as a contributing factor, we initially prepared and acquired 25 HeLa and 25 HEK-293 cells from one cell passage in five TMT10 analytical runs (proteoCHIP batch 1, supplemental Fig. S4). Clustering analysis of the 50 no-carrier single-cells from proteoCHIP batch 1 separated the two distinct cell types *via* the first two PCs (Fig. 4*D*; Kernel density estimation in PC1). Based on the two distinct clusters identified in the principal component analysis, we assigned the respective sample channels to two groups and evaluated the top differentially expressed proteins (Fig. 4, *D* and *E*). The single-cell proteome signatures define two distinct clusters consistent with the ground truth knowledge of these benchmarking samples (Fig. 4*D*). Additionally, numerous proteins observed in our dataset that appear to differentiate the two cell lines parallel the normalized expression data in the Human Protein Atlas (http://www.proteinatlas.org) (*i.e.*, brain acid soluble protein 1 (BASP1), FOLR1, H2AZ2, SLC7A5, UCHL1, and TMSB4X; Fig. 4*E*) (40). Interestingly, one of the top hits in HeLa cells, derived from a rare cervical adenocarcinoma, is the BASP1, which is downregulated in most tumor cell lines except some cervical cancer lines (Fig. 4*E*). Elevated levels of the tumor suppressor BASP1 is known to promote tumor growth and correlate with clinical aggressiveness in cervical cancer (41, 42).

As many SCP applications require analysis of larger sample cohorts, and to mimic typical sources of variability, we analyzed an additional 120 single cells from two distinct cell passages (proteoCHIP batches 2 and 3) for a total of 170 HeLa/HEK-293 no-carrier cells (*i.e.*, 85 HeLa and 85 HEK-293 cells, Fig. 4, *D* and *E* and supplemental Fig. S4). Importantly, the initial SCP analysis displayed in Figure 4, *D* and *E* represents one biological replicate with one cell passage, one proteoCHIP sample preparation workflow, and sequential MS-acquisition (Fig. 4, *D* and *E*). To increase potential technical variability and evaluate reproducibility of our proteoCHIP-based SCP sample preparation, six TMT10 HeLa/HEK-293 samples were prepared from distinct cell and buffer batches on two separate days and acquired with 1 week of acquisition break. In total, this combines three individual, biological replicates with three cell batches, three sample preparation workflows, and three acquisition days (supplemental Fig. S4). We identified 1912 distinct peptides across the 170 single cells with at least 70% quantitative data completeness without matching between runs or imputation (Fig. 4*F*). For this, we aggregated quantitative data of 17 analytical runs on the peptide level and filtered for reporter ion signal in at least 119 single cells. Again, we can clearly identify the two cell populations based on PC1 with an explained variance of 14.7% (Fig. 4*F*). Based on Kernel density estimation, we defined the two distinct clusters and found respective proteome characteristics to correspond to the 50-cell dataset and the Human Protein Atlas (Fig. 4, *E*–*G*) (40). Strikingly, despite cumulative missing values across 170 single cells, we identify CD44, a cell surface adhesion receptor expressed in many cancers and involved in metastasis, as differentially expressed (43). We are therefore confident to accurately distinguish and identify cell types based on quantitative proteome differences across 170 single cells using our proteoCHIP-based workflow (Fig. 4, *D*–*G*).

Interestingly, despite rigorous quality control, some HeLa and HEK-293 cells separated from their respective clusters when increasing cell numbers from the distinct proteoCHIP batches (Fig. 4, *D* and *F* and supplemental Fig. S5*D*). While we cannot exclude reduced sample overlap resulting from stochastic precursor selection and the concomitant reduction in analysis depth as a source of variability, we explored pathway associations of proteins that drive this separation. For this, we subjected proteins that were highly divergent in the “outlier” *versus* the “non-outlier” cells to pathway analysis (supplemental Fig. S5*D*). We found that more than 50% of those proteins are involved in metabolic regulation, most of which are less abundant in the “outlier” samples (supplemental Fig. S5*E*). Additionally, these “outlier” HeLa and HEK-293 single cells are attributed to one cell batch; we therefore suspect that not only the cell cycle, as shown by others but also metabolic regulation affects or may mask biological differences in the analysis and interpretation of SCP data (2, 12, 44, 45). Follow-up work with larger numbers of single cells will show the impact of differences in metabolism on the ability to explore proteome signatures of cellular subpopulations.

## Discussion

Here, we have demonstrated development and automation of SCP sample preparation using the proteoCHIP in conjunction with a commercial single-cell isolation and picoliter dispenser, the cellenONE. Our optimized protocol reduces digest volumes compared to previously published well-based techniques and is comparable to those successfully applied on modified glass slides (9, 12, 24, 33, 46). This not only minimizes chemical noise but the oil layer eliminates evaporation for constant enzyme and chemical concentrations. Next to the sample preparation efficiency, the specialized design of the proteoCHIP allows for automatic pooling of multiplexed samples to the proteoCHIP funnel using a standard benchtop centrifuge. This enables direct interfacing of the single-cell samples with a standard autosampler for LC-MS/MS analysis without the need for transfer or a specialized equipment (Fig. 1, *A*–*C*).

Our semiautomated sample preparation eliminates all manual sample handling resulting in comparable protein identifications and enhanced S/N of single cell reporter ions even at reduced or eliminated carrier (Fig. 1, *D* and *E*) (12, 24). This increases throughput by labeling single cells with all available TMT reagents and also improves the confidence of identifying peptides originating from single cells rather than the carrier (7). Despite the good correlation between individual samples (r = >0.85; Fig. 2, *A*–*D*) and exceptional data completeness of individual samples, the identification overlap between analytical runs is still subject to improvement (Fig. 2, *E*–*H*).

Additionally, we demonstrate that use of a somewhat wider precursor isolation window for analysis of multiplexed single-cell precursors does not significantly impact quantification accuracy or the ability to characterize differences between cell populations (29). In contrast to most high-input studies, we and others show that for single-cell input, relatively long chromatographic separation (*i.e.*, 60-min) in conjunction with FAIMS-based ion mobility separation and rapid cycle times reduce precursor co-isolation to a minimum (15, 29, 30, 31, 36). We speculate that most co-eluting precursors are below the limit of detection and therefore do not result in chimeric spectra (Fig. 3*A*). As expected, we demonstrate that especially at 10 and 100 ng peptide input, wider isolation windows increase identifications in part due to the ability to deconvolve and identify co-eluting precursors that give rise to chimeric spectra (Fig. 3, *A* and *B*). Ratio compression is a well-characterized disadvantage of TMT-based quantification at low and high input but does not affect the ability to define cellular subtypes, especially at low nanogram input (Figs. 3, *E*–*J* and 4, *D*–*G*) (19, 47). The increased ratio compression at higher inputs with wider isolation windows is illustrated by the reduced explained variance in PC1 (Fig. 3, *F*, *G*, *I* and *J*). Most interestingly, we observed increased explained variance with 2 m/z isolation window compared to 0.4 m/z at 1 ng total peptide input. This indicates that at lower peptide input level, wider isolation windows can be used to boost sensitivity without increasing interference, which is not the case for >10 ng peptide (Fig. 1, *E*–*J*). We speculate that this is due to the combination of relatively long gradients, FAIMS-based filtering, and limit of detection of current mass spectrometers when analyzing 100 pg peptides per sample. Concluding, the isolation window width or usage of nonisobaric single-cell approaches in conjunction with our highly efficient proteoCHIP-based sample preparation should therefore be carefully evaluated.

Importantly, we benchmark our proteoCHIP workflow for cell type–specific clustering of two human cell types with paralleling protein abundances to the Human Proteome Atlas for both cell types (Fig. 4, *D*–*G*) (40). Within this dataset, we observed that precursor stochasticity in such low-abundant samples results in sufficient sampling of abundant peptides. Our stringent quality metrics therefore drastically reduce the quantitative data and ultimately result in coalescent cell clusters (Fig. 4, *D* and *F*). Investigation of these “outlier” cells indicated that they are attributed to one proteoCHIP workflow (*i.e.*, cell passage) and were depleted for metabolically active proteins, which may have caused them to separate from the other cells (supplemental Fig. S5, *D* and *E*). To improve coverage and reduce missingness in large multiplexed SCP studies, recently described approaches leverage real-time search for MS2- or MS3-based quantification (32, 48). Based on that, we are convinced that our proteoCHIP sample preparation in conjunction with dedicated acquisition strategies will increase analysis depth to profile similar subpopulations based on their proteome at single-cell resolution. Moreover, the excellent reporter ion S/N across multiple samples and >75% data completeness per analytical run with present acquisition strategies leaves us confident that multiplexed data independent acquisition or more selective DDA workflows will further advance data quality (29, 32).

Overall, the integrated use of our proteoCHIP with the cellenONE achieves efficient SCP sample preparation and reduces detrimental peptide losses enabling in-depth characterization of diverse single cell samples. The high level of automation and commercial availability of all components should facilitate adoption of the approach by many proteomics laboratories.

## Data Availability

All mass spectrometry-based proteomics data have been deposited to the ProteomeXchange Consortium *via* the PRIDE partner repository with the dataset identifier [PXD025387].

## Supplemental data

This article contains supplemental data.

## Conflict of interest

D. H. (since 02/2022), A. S. and G. T. are employees of Cellenion. S. A. C. is a member of the scientific advisory boards of Kymera, PTM BioLabs, Seer and PrognomIQ. All other authors declare that they have no conflicts of interest with the contents of this article.
