# Supplementary material for: An Automated Nanowell-Array Workflow for Quantitative Multiplexed Single-Cell Proteomics Sample Preparation at High Sensitivity
Source: Mol Cell Proteomics. 2023 Oct 14;22(12):100665. doi: 10.1016/j.mcpro.2023.100665 (PMC10684380; doi:10.1016/j.mcpro.2023.100665)
Supplement: Supplementary file 1 [file mmc1.docx]

# Title

An automated nanowell-array workflow for quantitative multiplexed single-cell proteomics sample preparation at high sensitivity.

# Authors

Claudia Ctortecka^1,2,*,§^, David Hartlmayr^1,3*^, Anjali Seth^3^, Sasha Mendjan^4^, Guilhem Tourniaire^3^, Namrata D. Udeshi^2^, Steven A. Carr^2,§^ and Karl Mechtler^1,3,4,5,§^

* These authors contributed equally to this work.

# Affiliation

1 Research Institute of Molecular Pathology (IMP), Vienna BioCenter (VBC), Campus-Vienna-Biocenter 1, 1030 Vienna, Austria.

2 Broad Institute of MIT and Harvard, 415 Main Street, 02142 Cambridge, MA, USA.

3 Cellenion SASU, 60F avenue Rockefeller, 69008 Lyon, France.

4 Institute of Molecular Biotechnology of the Austrian Academy of Sciences (IMBA), Vienna BioCenter (VBC), Dr. Bohr-Gasse 3, 1030 Vienna, Austria.

5 The Gregor Mendel Institute of Molecular Plant Biology of the Austrian Academy of Sciences (GMI), Vienna BioCenter (VBC), Dr. Bohr-Gasse 3, 1030 Vienna, Austria.

# Running Title

The proteoCHIP for multiplexed single-cell proteomics

# ^§^Correspondence

Claudia Ctortecka	[cctortec@broadinstitute.org](mailto:Claudia.ctortecka@imp.ac.at)

Steven A. Carr	[scarr@broad.mit.edu](mailto:scarr@broad.mit.edu)

Karl Mechtler	[Karl.mechtler@imp.ac.at](mailto:Karl.mechtler@imp.ac.at)

**Supplemental Figure 1: Surface comparison reveals comparable peptide losses across tested materials.** Gravy index of hydropathy of bulk HeLa digest in glass vials (bulk), in-silico digested human FASTA (in-silico), HeLa cells prepared standard plastic ware (PP), the proteoCHIP (Teflon) and prepared in the proteoCHIP but transferred to a standard PCR vial for injection (Transfer) across all PSMs.

**Supplemental Figure 2: Peptides unique to carrier samples are lower in abundance than peptides that are shared or found uniquely in the no-carrier samples.** Log_10_ reporter ion S/N of peptide sequences that are shared or unique to the carrier or no-carrier samples for **(a)** TMT10 and **(b)** TMTpro. RI = reporter ion.

 **Supplemental Figure 3: At 1ng peptide input the majority of MS/MS give rise to only one PSM, minimizing the impact of wider isolation windows on average reporter ion S/N.** **(a)** Count of identified peptides per MS/MS scan per isolation window (0.4, 0.7, 1, 1.5, 2 or 3 m/z) of the two proteome-mix is shown at 1, 10 or 100 ng total peptide input. Colors indicate the number of peptides identified per MS/MS scan. **(b)** Distribution of average reporter ion S/N of conditions displayed in a. RI = reporter ion.

**Supplemental Fig. 4: Schematic illustration of HeLa/HEK-293 proteoCHIP experimental setup.** Three proteoCHIP batches of three biological replicates of HeLa and HEK-293 cells are collected and prepared on separate days. From proteoCHIP batch 1, six TMT10-plex samples were prepared each comprised of 8 single-cells (4 HeLa and 4 HEK-293 cells), a 20x carrier channel comprised of 10 HeLa and 10 HEK-293 cells, and an empty channel to compensate for isobaric interference of the 20x carrier channel, resulting in a total of 48 single-cells. Additionally, from proteoCHIP batch 1, five TMT10-plex samples were prepared each comprised of 10 single-cells (5 HeLa and 5 HEK-293 cells), resulting in a total of 50 single-cells without a carrier. From biological replicate 2, proteoCHIP batch 2 was prepared, including six TMT10-plex samples, each comprised of 10 single-cells (5 HeLa and 5 HEK-293 cells), resulting in a total of 60 single-cells without a carrier. From biological replicate 3, proteoCHIP batch 3 was prepared, including six TMT10-plex samples, each comprised of 10 single-cells (5 HeLa and 5 HEK-293 cells), resulting in a total of 60 single-cells without a carrier. The combination of all three proteoCHIP batches results in 170 single-cells without a carrier (85 HeLa and 85 HEK-293 cells). Across all three proteoCHIP batches and both, no-carrier and carrier samples we acquired a total of 218 single-cells (109 HeLa and 109 HEK-293 cells).

**Supplemental Figure 5: HeLa and HEK-293 cell type characterization of multiple cell batches is influenced by metabolically active proteins.** **(a)** Image of a HEK-293 and a HeLa cell during image-based cell sorting using the cellenONE®. **(b)** Log_10_ reporter ion intensity distribution across all channels for 20x sorted and no-carrier samples. **(c)** Distinct peptide sequence overlap of three HeLa/HEK-293 analytical runs with 20x carrier. **(d)** PCA with Kernel density estimates of 170 single-cells displayed in Figure 4f. Cells beyond 25th or 75th percentile ± 1.5*interquartile range of PC2, separating from the distinct HeLa (pink) and HEK-293 (blue) clusters are considered ‘outliers’ (red). **(e)** Log_10_ reporter ion intensity distribution for proteins that are associated with metabolism across all channels are decreased for ‘outlier’ versus ‘non-outlier’ single-cells. RI = reporter ion.
